# Enhanced Chemiresistive Sensor Performance through Superior Accessibility to Metal Complex Sites via Triptycene-Skeleton Coordination Nanosheets

**DOI:** 10.1021/acsami.6c00170

**Published:** 2026-04-01

**Authors:** Hiroaki Maeda, Yuta Sudo, Kenji Takada, Naoya Fukui, Hiroshi Nishihara

**Affiliations:** † Research Institute for Science and Technology, 26413 Tokyo University of Science, 2641, Yamazaki, Noda, Chiba 278-8510, Japan; ‡ Graduate School of Science and Technology, Tokyo University of Science, 2641 Yamazaki, Noda, Chiba 278-8510, Japan

**Keywords:** coordination nanosheets, chemiresistive sensors, humidity, metal complexes, accessibility

## Abstract

Conventional chemiresistive sensors based on metal oxide semiconductors offer high sensitivity, fast response, and low cost. However, the requirement for high-temperature operation is a major issue. Recently, conductive π-conjugated coordination nanosheets composed of metal ions and π-conjugated planar ligands have attracted attention as active materials for chemiresistive sensors operating at room temperature (RT). However, their tendency toward multilayer formation, driven by strong interlayer interactions, impedes access to metal complexes (active sites), thereby reducing their performance. In this study, we synthesized a composite (NiHATT/CNT) consisting of a coordination nanosheet (composed of nickel ions and the triptycene-based three-branched ligand, 2,3,6,7,14,15-hexaaminotriptycene (HATT)) and a carbon nanotube (CNT). We evaluated the humidity response of the chemiresistive sensors using NiHATT/CNT as the active material. The introduction of the triptycene skeleton allowed the square-planar metal complexes to form perpendicular to the two-dimensional surface, thereby allowing analytes to easily access the active sites. Consequently, the NiHATT/CNT chemiresistive sensor exhibited a significantly greater response than the sensor using an active material consisting of a conventional coordination nanosheet with a planar π-conjugated ligand. This demonstrates that the chemical design that provides high accessibility to the metal complexes in NiHATT by introducing a triptycene skeleton enhances the sensing performance of the NiHATT/CNT composite.

## Graphical abstract

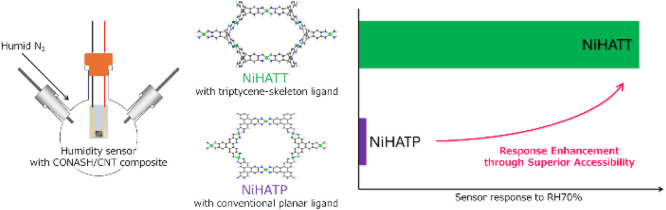

## Introduction

Highly sensitive and selective sensor elements are anticipated to be used in a variety of applications, including the early detection of hazardous and flammable gases, monitoring of air pollutants and environmentally harmful substances, and noninvasive medical diagnostics. Chemiresistive sensors detect chemical substances by measuring changes in electrical resistance, offering high sensitivity, fast response, and low cost.^1^ Conventional chemiresistive sensors often use metal oxide semiconductors (MOSs) as active materials. However, sufficient sensitivity mandates high-temperature operation, which leads to complex device structures, high power consumption, device degradation, and low selectivity.^2,3^ To address these issues, new active materials have been developed. One promising active material is two-dimensional materials, such as coordination nanosheets or two-dimensional metal–organic frameworks (MOFs), obtained by arranging π-conjugated planar ligands via square-planar metal complexes.^2,4–7^ π-Conjugated coordination nanosheets possess unique properties, including electrical conductivity, a large surface area, and a porous structure. These properties facilitate smooth electron and mass transport, enabling the fabrication of chemiresistive sensors with high sensitivity and fast response during operation at room temperature.^8–10^ Furthermore, high selectivity can be achieved through interactions with target substances.^11–14^ However, these π-conjugated coordination nanosheets readily stack, leading to multilayer formation via interlayer interactions driven by their planar chemical structure (Figure 1A). This could raise concerns that metal complexes, as active sites, may be shielded by neighboring layers, leading to reduced performance. Recently, research has focused on the synthesis of coordination nanosheets using a triptycene-based three-branched ligand instead of a π-conjugated planar ligand to overcome these issues (Figure 1B).^15–17^ The introduction of the triptycene skeleton allowed the square-planar metal complexes to form perpendicular to the two-dimensional surface. This chemical structure weakens the interlayer interactions and provides high accessibility to the metal complex sites.^15–18^ This enables guest substances (e.g., reactants, ions, and molecules) to easily access the metal complexes (active sites), thereby potentially improving the performance of the electrode material. Recently, we reported the synthesis of NiHATT, a coordination nanosheet consisting of a ligand with a triptycene skeleton, 2,3,6,7,14,15-hexaaminotriptycene (HATT), and nickel ions, via a gas–liquid interfacial reaction and electrochemical oxidation and achieved simple exfoliation to produce the thin sheets.^18^ Furthermore, we demonstrated that NiHATT exhibits superior electrocatalytic performance for hydrogen evolution reactions compared to conventional coordination nanosheets consisting of π-conjugated planar ligands. In addition, research has been conducted on the application of nanosheets with a triptycene skeleton in gas adsorption and separation,^19^ electrode catalysts,^15–17^ electrochemical sensors,^20^ secondary batteries,^21,22^ and electronics and spintronics.^23^ However, there are only a limited number of reports on experimental comparisons of the performance of coordination nanosheets using conventional π-conjugated planar ligands, and the advantages of chemical structure manipulation through the introduction of a triptycene skeleton have not yet been fully demonstrated.^15,17^

**Figure 1:**
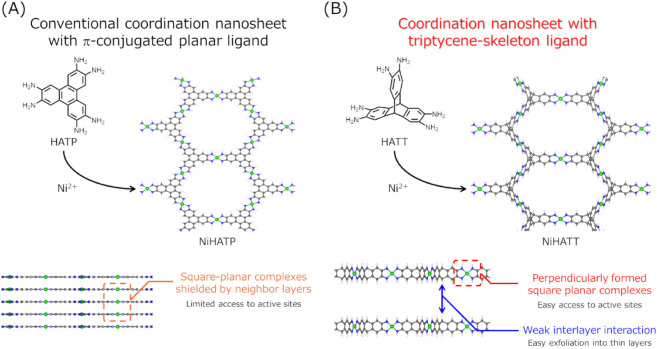
Chemical structures and structural features of coordination nanosheets with (A) representative conventional π-conjugated planar ligand (2,3,6,7,10,11-hexaaminotriphenylene, HATP) and (B) hexaaminotriptycene (HATT).

In this study, NiHATT was used as the active material in a chemiresistive sensor. To enhance the conductivity of the active material, NiHATT was synthesized in the presence of conductive single-walled carbon nanotubes (CNTs) and obtained as a composite material. The composite, NiHATT/CNT, was cast onto an interdigitated gold electrode to fabricate the sensor. The impedance change of the sensor in response to humidity changes was recorded to evaluate the response performance of NiHATT/CNT. Similarly, the response performance of NiHATP/CNT, a composite of carbon nanotubes and a coordination nanosheet composed of the π-conjugated planar ligand, 2,3,6,7,10,11-hexaaminotriphenylene (HATP), was evaluated. NiHATT/CNT exhibited a greater response to humidity changes than NiHATP/CNT, indicating that NiHATT/CNT is a superior active-sensing material. These results suggest that increasing the accessibility of active sites by introducing a triptycene skeleton can enhance the performance of coordination nanosheets in sensing material applications. Furthermore, a comparison of the humidity responsivity of the NiHATT-CNT mixture prepared by simply mixing NiHATT and CNT and Ni­(oPDI)_2_/CNT (a composite of the mononuclear complex and CNT) showed that favorable interactions between a coordination nanosheet and CNT contributed to the high responsivity.

## Results and Discussion

Figure 2A shows the synthetic procedure for the NiHATT/CNT composites. Under an Ar atmosphere, Ni­(OAc)_2_·4H_2_O (3.0 mg, 12 μmol), HATT·6HCl (9.0 mg, 8 μmol), and single-walled carbon nanotubes (CNT, 0.5, 1, 5, or 10 mg) were added to a 0.1 M NH_3_ methanol solution (20 mL). Reaction vials were capped with septum caps. Because the complexation between nickel ions and HATT requires oxidation, the mixture was stirred overnight at room temperature, with air naturally diffusing into the reaction vessel through a needle piercing the septum. The resulting dispersion was centrifuged, washed with methanol, and acquired as 2 mL of the NiHATT/CNT-*X* (*X* = 0.5, 1, 5, 10) methanol dispersion, where *X* is the mass of carbon nanotubes used in the synthesis. The NiHATT-CNT mixture obtained by stirring overnight in methanol with 1 mg of CNT and NiHATT, and the CNT stirred overnight in a 0.1 M NH_3_ methanol solution, was also prepared as reference materials.

**Figure 2:**
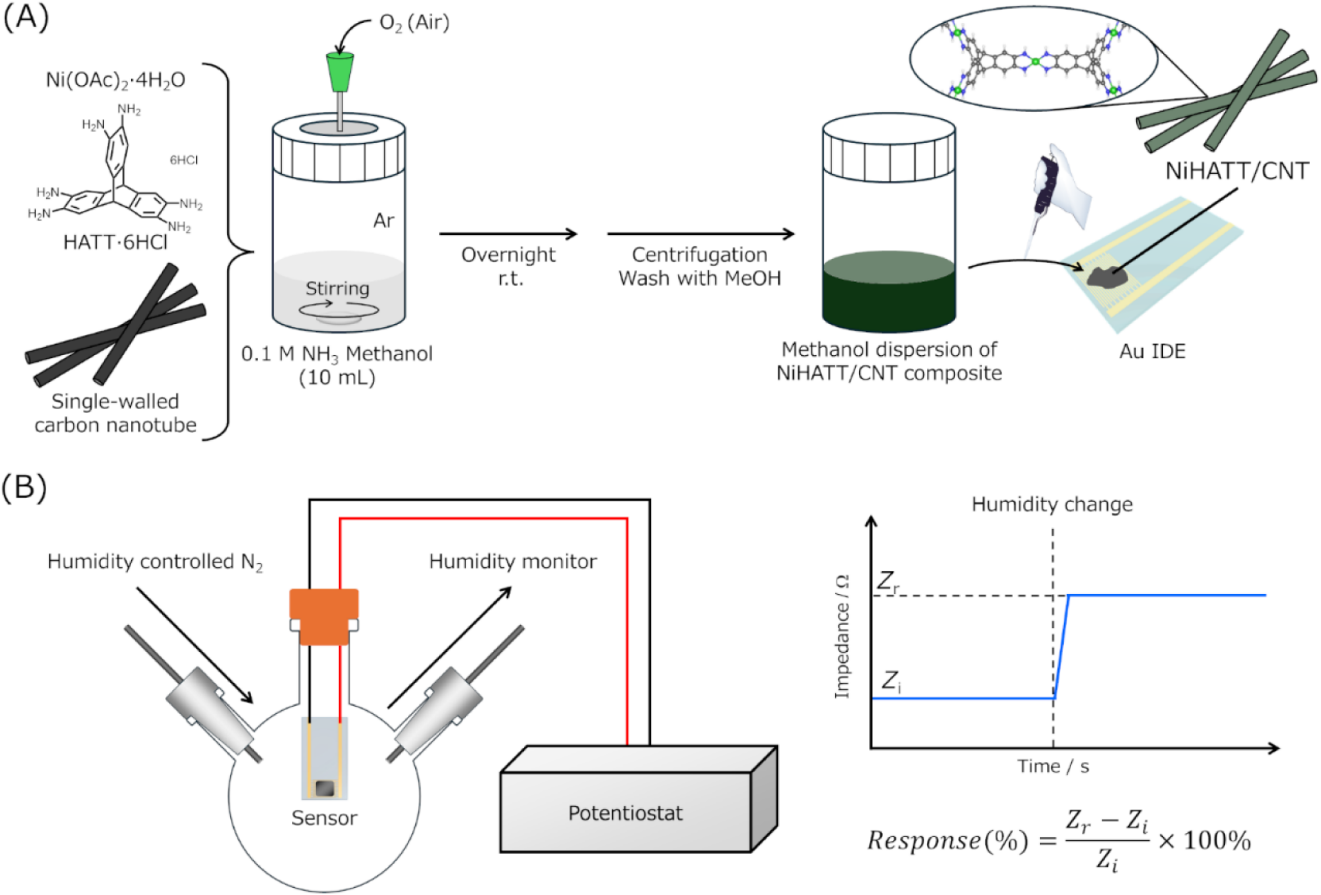
(A) Synthetic procedures for NiHATT/CNT composites and the preparation of sensor devices. (B) Illustration of humidity response sensor performance evaluation.

The microscopic and spectroscopic results for NiHATT/CNT-1 are shown in Figure 3 as representative data. Those of NiHATT/CNT-*X* (*X* = 0.5, 5, and 10) and NiHATT-CNT mixtures are shown in the Supporting Information (Figures S1 and S2). Scanning electron microscopy (SEM) observations showed that the material was an integrated, fibrous structure with a flat or powdery appearance (Figure 3A). EDS elemental mapping revealed that carbon, nitrogen, and nickel were distributed throughout the observed structures. The presence of C, N, and Ni in the NiHATT/CNT-*X* and NiHATT-CNT mixtures was confirmed (Figures S1A, D, G and S2A). TEM also revealed the powdery structure of NiHATT/CNT-1 integrated with a fibrous structure (Figure 3B). However, a fine periodic structure was not clearly observed in high-resolution TEM observations, suggesting the low crystallinity of NiHATT/CNT-1 (Figure S3A). High-resolution STEM/EDS mapping revealed that Ni and N are distributed in the fibrous structure derived from the CNTs, suggesting that the CNTs are covered with NiHATT (Figures S3B). These results indicate the formation of a NiHATT/CNT composite with integrated NiHATT and CNT. On the other hand, TEM images of the NiHATT-CNT mixture clearly showed that fibrous structures and round-shaped structures derived from CNT and NiHATT, respectively, were distributed separately (Figures S2B and C). This observation reveals that the simple mixing of CNT and NiHATT does not yield a NiHATT/CNT composite integrated with the two materials. In NiHATT synthesis in the presence of CNTs, HATT ligands with a triptycene backbone can be adsorbed onto the sidewalls of the CNTs via π–π stacking interaction.^24–26^ The formation of NiHATT proceeds from adsorbed HATT, yielding a NiHATT/CNT composite with a π–π interaction between the vertical π-conjugated system of NiHATT and the π-conjugated surface of CNTs. Powder X-ray diffraction (XRD) did not show an obvious pattern, suggesting low crystallinity and low stacking orientation of NiHATT/CNT-1 (Figure S3C). A potential reason for the low crystallinity is the formation of ring structures smaller than the ideal hexagonal structure due to the high structural permissibility of the HATT ligand.^27^ The structure with smaller pore sizes than the ideal structure may result in disadvantages in terms of mass transport. In addition, the metal complex sites are distorted from square-planar geometry. Although these structural challenges remain, the introduction of the triptycene skeleton allows NiHATT to form the distorted square-planar metal complexes perpendicular to the two-dimensional surface. Hence, NiHATT can still provide high accessibility to the active site. The IR and Raman spectra provide chemical bonding information. NiHATT and NiHATT/CNT-1 exhibited similar IR spectra because the CNT did not exhibit any obvious IR-active peaks (Figure 3C). The peaks at 1000–1650, 2920 are assigned to C=C and C–N, and C–H stretching vibrations, respectively. The broad peak around 3400 cm^–1^ in the IR spectra in Figure 3C originates from the N–H stretching of NiHATT. In contrast, the Raman spectrum of NiHATT/CNT includes peaks observed for both NiHATT and CNT, indicating that NiHATT and CNT exist at the same location (Figure 3D). The peaks at 470, 620, and 1200–1700 cm^–1^ derive from the Ni–N stretching, N–Ni–N bending, and C=C stretching vibrations in NiHATT, corresponding to our previous report.^18^ The peaks at 160–220, 1590, and 2670 cm^–1^ can be assigned to the radial breathing mode (RBM), G-band, and 2D band in CNT, respectively. The diameter of CNT was estimated as ca. 1.3 nm from the peak top of RBM observed at ca. 190 cm^–1^.^28^ All spectra of NiHATT/CNT-1 recorded at multiple points on the sample showed peaks from both NiHATT and CNT, indicating that they coexist throughout the composite (Figure S3D). In contrast, most of the Raman spectra of the NiHATT-CNT mixture recorded at multiple points show only the peaks derived from CNTs or significantly weak peaks derived from NiHATT, suggesting the uneven presence of NiHATT and CNT in the sample (Figure S2D). Furthermore, the IR and Raman spectra of NiHATT/CNT-*X* (*X* = 0.5, 5, and 10) showed a clear dependency on the quantity of CNTs added during the synthesis. NiHATT/CNT-0.5 showed obvious peaks derived from NiHATT in the IR and Raman spectra, and the intensity of these peaks drastically decreased with an increasing quantity of CNTs (Figures S1B, C, E, F, H, and I). X-ray photoelectron spectra provided information on the chemical states of Ni and N in the samples. Both NiHATT and NiHATT/CNT-1 showed the peaks derived from Ni 2p_3/2_, Ni 2p_1/2_, and N 1s at 855.8, 873.3, and 399.6 eV, respectively (Figure 3E). The broad satellite peaks were also observed at 862.2 and 880.3 eV in the Ni 2p spectra. The NiHATT-CNT mixture exhibited similar spectra, suggesting that the NiHATT/CNT-1 and NiHATT-CNT mixtures had the same chemical state as NiHATT, even in the presence of CNTs (Figure S2E). These results reveal that NiHATT/CNT composites were synthesized by integrating NiHATT and CNTs using a facile procedure.

**Figure 3:**
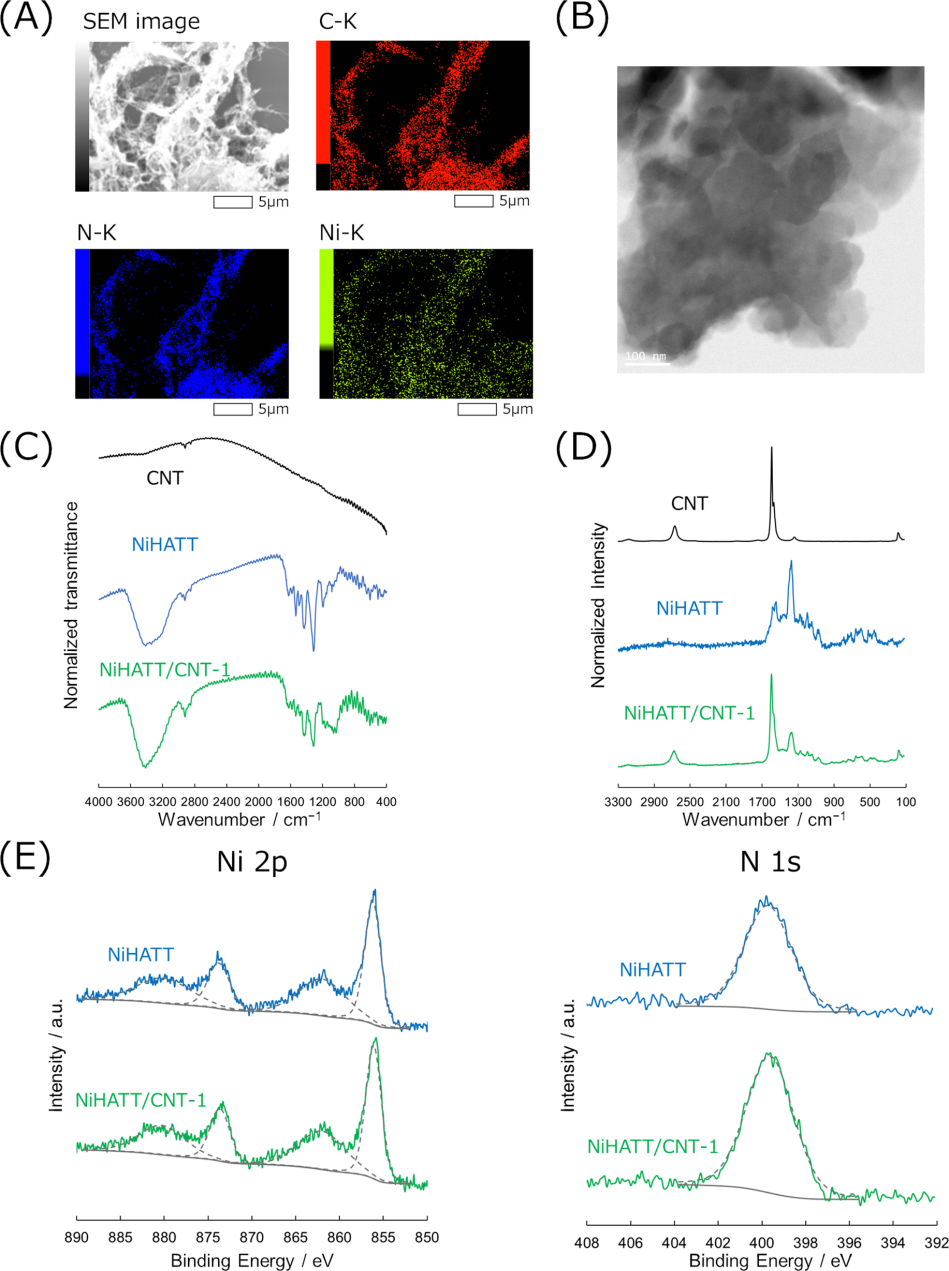
(A) SEM image, EDS mapping of C, N, Ni, and (B) TEM image of NiHATT/CNT-1. (C) IR and (D) Raman spectra of CNT, NiHATT, and NiHATT/CNT-1. (E) X-ray photoelectron spectra of NiHATT and NiHATT/CNT-1 at Ni 2p and N 1s regions. Dashed and solid gray lines represent fitting curves and the background line, respectively.

The humidity responses of the NiHATT/CNT-1 composite, NiHATT-CNT mixture, and CNT were evaluated. Sensor electrodes were prepared by casting 10 μL of the respective methanol dispersions onto interdigitated Au electrodes (electrode width: 10 μm; electrode distance: 5 μm), followed by drying (Figure 2A). The sensor electrodes were placed in an N_2_-filled vessel, with the temperature maintained at 25 °C using an incubator (Figure 2B). An alternating current (AC) voltage with an amplitude of 10 mV at 1000 Hz was applied at a DC bias of 0 V to the sensor to measure its initial impedance under dry N_2_ conditions. Then, the N_2_ gas with a relative humidity (RH) of 10% was introduced into the vessel to record the impedance changes of the sensor. After maintaining these conditions for 3 min, dry N_2_ was reintroduced into the vessel to reset the sensor. The impedance changes at RH30%, 50%, and 70% were recorded in the same manner to evaluate the response abilities of the sensors to humidity changes. In addition, the repeated response abilities of the sensors were evaluated by the alternative introduction of N_2_ gases with RH10% and 70% every 3 min into the vessel. The response intensity was calculated as a ratio of the impedance difference before and after humidity change (*Z*_r_ – *Z*_i_) and the *Z*_i_ (Figure 2B). Figure 4A and B display representative changes in the real part of impedance for each material and their average response intensities at RH10, 30, 50, and 70%. All sensors exhibited insignificant changes in phase up to 0.3° at a frequency of 1000 Hz under humidity change, suggesting that the capacitance or inductance factors did not significantly contribute to the impedance change induced by the humidity change (Figure S4B). Hence, the real part of the impedance can be approximated to reflect the total impedance. While the sensors using the NiHATT-CNT mixture and CNT exhibited initial impedances of 7.5 ± 1.2 Ω and 10.2 ± 1.5 Ω, respectively, under dry N_2_. Despite CNT showing higher impedance than the NiHATT-CNT mixture, this may be attributed to contact resistance resulting from the measurement method used in this study. On the other hand, the NiHATT/CNT-1 sensor showed an impedance 1 order of magnitude larger at 200 ± 140 Ω. This difference may be because CNTs are mainly responsible for electron transport in the NiHATT-CNT mixture and CNT, whereas NiHATT, which has a lower electrical conductivity than CNTs, is present in the electron transport pathway in NiHATT/CNT-1. The sensors with the NiHATT-CNT mixture and CNT exhibited negligible changes in response to humidity, with only a 0.2% change at RH70%. In contrast, the NiHATT/CNT-1 sensor showed significant impedance changes accompanied by a change in the humidity level; the impedance increased with increasing humidity and decreased when dry N_2_ was introduced (Figure 4A). Furthermore, the response intensity of NiHATT/CNT-1 proportionally increased with the humidity level and reached 30% under the RH70% condition (Figure 4B). The response and recovery times at RH 10% were determined to be 5 ± 0.8 s and 34 ± 8 s, respectively (Figure S5). The response abilities of NiHATT/CNT-1 are comparable to those of other advanced MOF-based and conjugated-polymer-based impedimetric humidity sensors.^29–31^ In the repeated response test, the sensors with the NiHATT-CNT mixture and CNT responded insignificantly to humidity changes. In contrast, the sensor with NiHATT/CNT-1 reversibly responded to the alternating humidity change between RH10% and 70%, with a maintained response intensity of approximately 15% (Figure 4C and D). These results indicate that NiHATT is an active material for humidity sensing and that favorable interactions between NiHATT and CNTs are essential for producing sensors with high response capabilities. The possible interaction is a π–π stacking between the vertical π-conjugated system of NiHATT and the π-conjugated surface of CNTs. The presence of water molecules induces a change in the strength of the π–π interaction between NiHATT and CNT, resulting in an alteration of the electrical conductivity of NiHATT/CNT-1.

**Figure 4:**
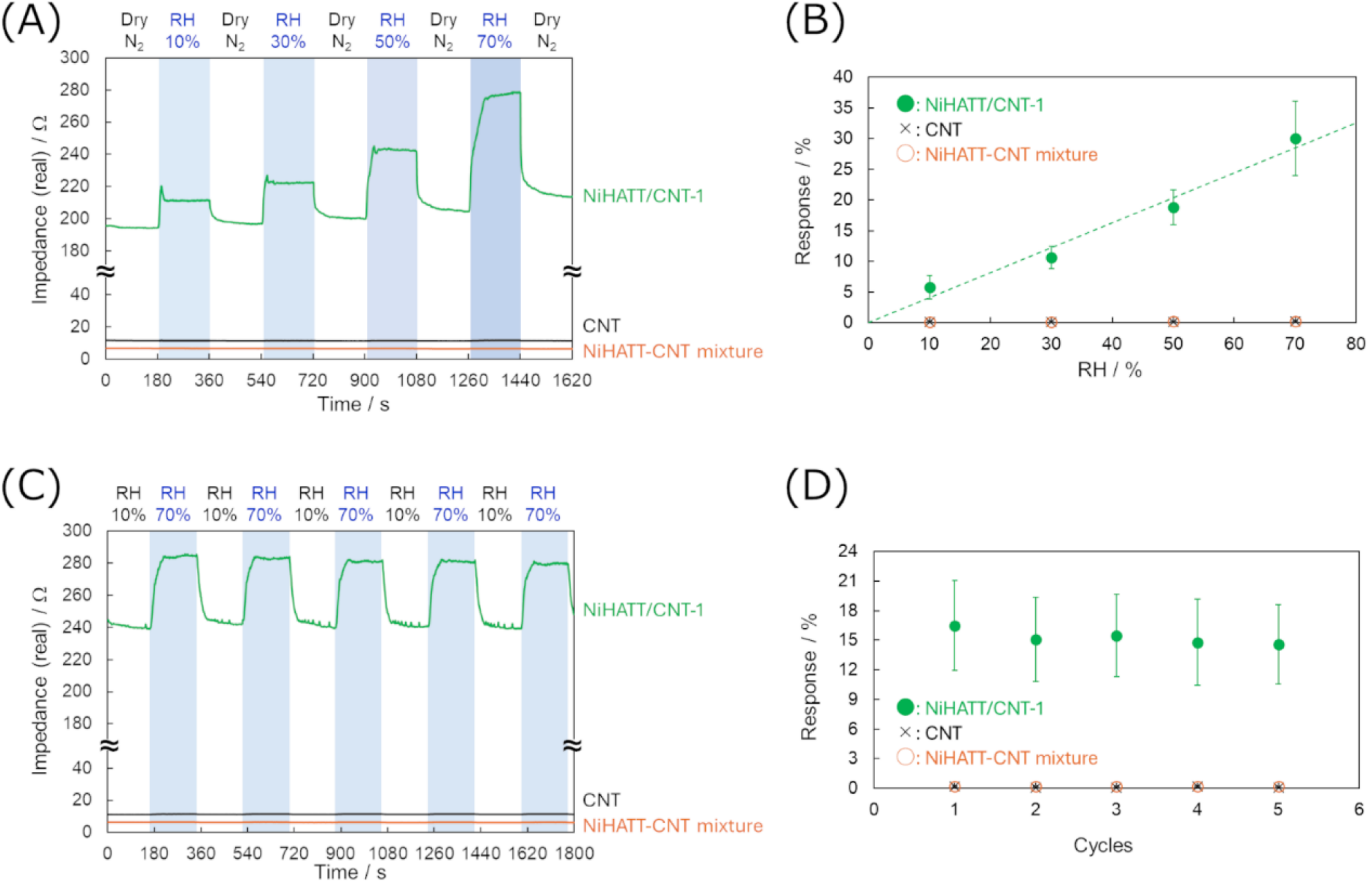
Sensing performance of chemiresistive sensors with NiHATT/CNT-1 (green), NiHATT-CNT mixture (orange), and CNT (black) for the detection of relative humidity (RH). (A) Impedance responses upon exposure to different RH levels in the range of 10–70%, and (B) the response intensities at each RH level. (C) Impedance responses upon repeated exposure to RH10% and 70%, and (D) the response intensities at each cycle.

The dependence of the humidity response abilities of the NiHATT/CNT-*X* composites on the quantity of CNT added during synthesis was also evaluated in the same manner. All the composites showed significant impedance changes in response to humidity changes, with a greater response at higher humidity (Figure 5A). In addition, reversible responses to humidity changes were observed in repeated tests (Figure 5B). In contrast, the response intensity decreased with increasing CNT content, *X* (Figure 5C). The initial impedance values of the sensors with NiHATT/CNT-*X* under dry N_2_ conditions also decreased with increasing *X*. The impedance of 11.7 ± 1.4 Ω for NiHATT/CNT-10 was almost equal to that of the sensor with CNT (10.2 ± 1.5 Ω) (Figure S6). The increase in CNT content decreased the proportion of NiHATT in the composites as an active sensing material. It increased the contribution of electron transport through the CNTs, leading to a depression in the humidity response. Although NiHATT/CNT-0.5 exhibited an average response intensity similar to that of NiHATT/CNT-1, the deviation was strongly dependent on the prepared sensor devices. Therefore, NiHATT/CNT-1 is a suitable material for humidity sensing owing to its high response intensity and good reproducibility of the device performance.

**Figure 5:**
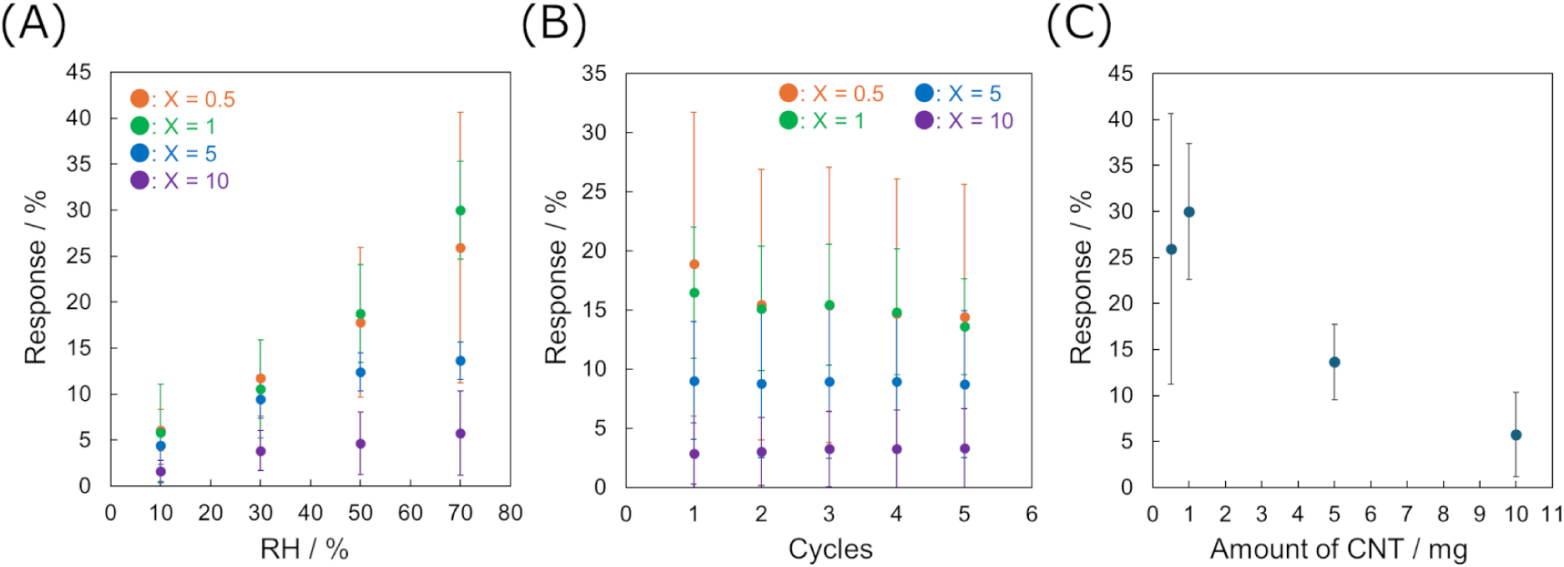
(A) Response intensities upon exposure to different RH levels in the range of 10–70%, and (B) repeated exposure to RH10% and 70% of chemiresistive sensors with NiHATT/CNT-*X* composites (*X* = 0.5, 1, 5, and 10). (C) Relation between response intensities upon exposure to RH70% and the amount of CNT added in the synthesis of NiHATT/CNT-*X* composites.

To clarify the contribution of the vertically formed square-planar NiN_4_ complex units in NiHATT to the sensing properties, we evaluated the humidity response abilities of the conventional coordination nanosheet with a π-conjugated planar ligand, NiHATP, and the metal complex bis­(*o*-phenylenediimine) (Ni­(oPDI)_2_), which is the motif of NiHATT and NiHATP. NiHATP, composed of nickel ions and 2,3,6,7,10,11-hexaaminotriphenylene (HATP), is a two-dimensional planar coordination nanosheet with pores similar in size to those of NiHATT (∼2 nm). Hence, we selected NiHATP as a comparison material for NiHATT because the comparison of humidity-sensing performance between NiHATT and NiHATP is suitable for demonstrating the advantage of the vertically formed square-planar complexes in NiHATT while eliminating the influence of differences in pore size. NiHATP/CNT-1 and Ni­(oPDI)_2_/CNT-1 composites were synthesized using procedures similar to those used for NiHATT/CNT-1 (see details in the Experimental Section). SEM observation and EDS mapping of NiHATP/CNT-1 revealed the presence of powdery materials on the fibrous structures with a uniform distribution of Ni and N (Figure S7A). The powder XRD showed the peaks at 2θ = 4.82, 9.64, 12.8, 16.7, and 27.6°, derived from the periodicity of NiHATP, revealing that NiHATP/CNT-1 contains crystalline NiHATP domains with the hexagonal unit cell of *a* = *b* = 21.1 Å and *c* = 3.2 Å (Figure S7B).^32^ Although NiHATP/CNT-1 and NiHATP showed similar IR spectra because CNT is IR inactive, the Raman spectrum of NiHATP/CNT-1 contains the peaks derived from both NiHATP and CNT, showing their coexistence in the composite (Figure S7C and D). XPS showed peaks at 855.2, 872.8, and 399.5 eV, corresponding to Ni 2p_3/2_, Ni 2p_1/2_, and N 1s, respectively, for both samples (Figure S7E). SEM and EDS mapping of Ni­(oPDI)_2_/CNT-1 revealed the coexistence of CNT and plate-shaped materials containing Ni and N (Figure S8A). IR and Raman spectra of Ni­(oPDI)_2_/CNT-1 and Ni­(oPDI)_2_ also revealed the presence of Ni­(oPDI)_2_ and CNT in the Ni­(oPDI)_2_/CNT-1 composite (Figure S8B and C). XPS spectra of Ni­(oPDI)_2_/CNT-1 and Ni­(oPDI)_2_ exhibited peaks at 855.4 and 872.7 eV derived from Ni 2p_3/2_ and Ni 2p_1/2_, respectively (Figure S8D). In the N 1s region, Ni­(oPDI)_2_ showed two peaks at 397.6 and 399.8 eV, while Ni­(oPDI)_2_/CNT-1 exhibited them at 398.0 and 399.9 eV. These peaks were assigned to nitrogen atoms in the quinoid (C=N) and benzoid (C–N) structures, respectively.^33–35^ The N 1s spectra of NiHATT/CNT-1 composite, NiHATT-CNT mixture, and NiHATP/CNT-1 composite only showed one peak around 399.6 eV, suggesting that the nitrogen atoms in these materials primarily take benzoid structures (Figures 3E, S2E and S7E). The difference in the intensity ratio of these peaks between the two samples can be explained by the fact that the samples exhibit different oxidation states due to electronic interactions between the Ni­(oPDI)_2_ complex and the CNT via π–π stacking.^36,37^ The presence of the Ni­(oPDI)_2_ complex was confirmed by ^1^H NMR spectroscopy (Figure S9). These results indicate that the desired composites NiHATP/CNT-1 and Ni­(oPDI)_2_/CNT-1 were obtained.

Their humidity response abilities were evaluated using the same procedure as that for NiHATT/CNT-1. The impedance values of sensors with NiHATP/CNT-1 and Ni­(oPDI)_2_/CNT-1 under dry N_2_ atmosphere were 7.3 ± 0.9 Ω and 14 ± 3 Ω, respectively, close to that of the sensor with CNT (10.2 ± 1.5 Ω), suggesting that the CNT works as the main electron transfer pathway. Both NiHATP/CNT-1 and Ni­(oPDI)_2_/CNT-1 exhibited weak responses to humidity changes, with 1.1% and 3.8% increases at 70% RH, respectively (Figure 6A and B). Their reversible responses to the alternating humidity changes between RH10% and 70% were also small: 0.8% for NiHATP/CNT-1 and 1.6% for Ni­(oPDI)_2_/CNT-1 (Figure 6C and D). The response ability of NiHATT/CNT-1 was superior to their performances, with a response intensity of 30% under the RH70% condition and 15% under the alternating humidity changes between RH10% and 70%. These results indicate that the significant contribution of vertically formed NiN_4_ complex sites in NiHATT enhances the sensing ability. Furthermore, the formation of a network structure by HATT ligands can improve the interaction with CNTs and the performance of the active materials, rather than using Ni­(oPDI)_2_ mononuclear complexes.

**Figure 6:**
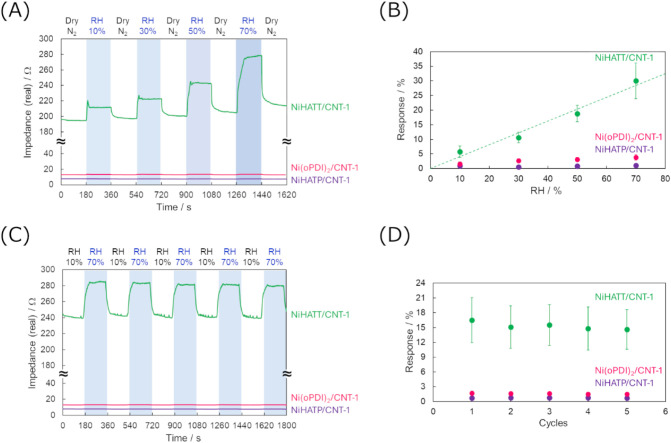
Sensing performance of chemiresistive sensors with NiHATT/CNT-1 (green), NiHATP/CNT-1 (purple), and Ni­(oPDI)_2_/CNT-1 (pink) for the detection of relative humidity (RH). (A) Impedance responses upon exposure to different RH levels in the range of 10–70%, and (B) the response intensities at each RH level. (C) Impedance responses upon repeated exposure to RH10% and 70%, and (D) the response intensities at each cycle.

## Conclusion

In summary, a NiHATT/CNT composite consisting of a triptycene-ligand-based coordination nanosheet (NiHATT) and CNT was synthesized. The humidity-response performance of the sensor as a sensing material for chemiresistive sensors was also evaluated. NiHATT/CNT showed a humidity response of 30% at RH 70% and a reversible response of approximately 15% for repeated humidity changes between RH 10% and 70%. This response was superior to those of two comparative composites: NiHATP/CNT, which uses coordination nanosheets with conjugated planar ligands, and Ni­(oPDI)_2_/CNT, which uses mononuclear complexes. These results indicate that NiHATT is a promising sensing material for chemiresistive sensors, offering a high detection capability. Furthermore, the excellent response properties are attributed to the chemical structure of NiHATT, which provides high accessibility to the active sites and favorable interactions with the CNTs. These findings will contribute to the development of more sensitive, low-power chemiresistive sensors based on 2D materials.

## Experimental Section

### Materials

Hexaaminotriptycene hexahydrochloride (HATT·6HCl) was synthesized according to the literature^38^ or purchased from BLD Phamatech. Single-walled carbon nanotube (CNT) purchased from Aldrich was ultrasonically dispersed in a mixture of concn HCl aqueous solution and MeOH to remove the metallic residues. The treated CNTs were collected via centrifugation, rinsed with MeOH, and dried under vacuum. The chemical reagents and organic solvents were purchased from commercial sources and used without further purification. Interdigitated gold electrodes (AuIDE-10/5) were purchased from Geomatech, Inc.

### Equipment

Scanning electron microscopy and energy-dispersive X-ray spectroscopy were performed using a JCM-7000 NeoScope (JEOL). Powder X-ray diffraction was performed using a Rigaku MiniFlex600-C diffractometer. X-ray photoelectron spectroscopy (XPS) was conducted using a PHI 5000 VersaProbe III (ULVAC-PHI) instrument with a monochromated Al Kα (15 kV, 25 W) X-ray source. The spectra were analyzed using MultiPak software, and the binding energy was calibrated using a Si 2p peak at 99.2 eV. Raman spectra were collected using an NRS-5500 (JASCO) instrument with a 532 nm excitation laser. IR spectra of KBr-pelletized samples were recorded using an FT/IR-6100 spectrometer (JASCO) under vacuum. Transmission electron microscopy (TEM) was performed at an accelerating voltage of 200 kV using a JEM-ARM200F microscope (JEOL). Humidity control for the sensing performance evaluation was conducted using AHCU-2 (Kits Microfilter Inc.). Impedance data were collected using HZ-4S (Hokuto Denko, Inc.). ^1^H NMR spectra were recorded using an ECZ-400S spectrometer (JEOL). The 3D chemical structures of NiHATT and NiHATP were drawn using VESTA.^39^

### Synthesis of Composites of Coordination Nanosheets and CNTs

Nickel acetate tetrahydrate (3.0 mg, 12 μmol), HATT·6HCl (9.0 mg, 8 μmol), and SWCNT (0.5, 1, 5, or 10 mg) were mixed in 0.1 M NH_3_ methanol (10 mL) under an argon atmosphere. The reaction vial was then capped with a septum. The reaction mixture was stirred overnight at room temperature, and air was introduced through a needle. The formed NiHATT/CNT-*X* composite (*X* indicates the amount of CNT) was collected as a 2 mL methanol dispersion after centrifuging and rinsing with methanol (twice). NiHATP/CNT-1 and Ni­(oPDI)_2_/CNT-1 composites were prepared by the same procedure, except using hexaaminotriphenylene hexahydrochloride (HATP·6HCl, 8.6 mg, 8 μmol) and *o*-phenylenediamine (5.3 mg, 24 μmol) as ligands, respectively.

### Synthesis of NiHATT, NiHATP, and Ni­(oPDI)_2_

NiHATT, NiHATP, and Ni­(oPDI)_2_ were synthesized according to the corresponding synthesis methods for composites without the addition of CNT. NiHATT and NiHATP were characterized by FT-IR, Raman spectroscopy, powder XRD, and XPS. Ni­(oPDI)_2_ was characterized using FT-IR, Raman spectroscopy, XPS, and ^1^H NMR. ^1^H NMR (400 MHz, DMSO-*d*_6_) δ 8.81 (s, 4H), 6.96 (dd, 4H), 6.67 (dd, 4H) (Figures S8 and S9).^40^

### Evaluation of Humidity-Sensing Performance

Sensors were prepared by dropping 10 μL of the NiHATT/CNT-*X*, NiHATP/CNT-1, or Ni­(oPDI)_2_/CNT-1 composites in methanol onto the Au IDEs, followed by drying under vacuum. The sensing electrodes are connected to a potentiostat and placed in a glass vessel. The desired relative humidity in the vessel was achieved by flowing humid nitrogen gas through a humidity controller. The impedance change of the sensor electrodes was measured by applying a 1000 Hz AC voltage at 0 V. The temperature during the measurements was maintained at 25 °C using an incubator. The sensing performance of each active material was evaluated using three to five independent sensors to confirm reproducibility.

## Supplementary Material



## Notes

Characterization of NiHATT/CNT-*X* (*X* = 0.5, 5, 10) (Figure S1); characterization of NiHATT-CNT mixture (Figure S2); TEM images, EDS mapping, XRD, and Raman spectra of NiHATT/CNT-1 (Figure S3); impedance and phase responses upon exposure to different RH levels in the range of 10–70% and repeated exposure to RH10% and 70% of chemiresistive sensors with NiHATT/CNT-*X* composites (*X* = 0.5, 1, 5, and 10) (Figure S4); response and recovery time of NiHATT/CNT-1 at RH10% (Figure S5); impedance of chemiresistive sensors with NiHATT/CNT-*X* composites (*X* = 0.5, 1, 5, and 10) under a dry nitrogen atmosphere (Figure S6); SEM image, EDS mapping, XRD, IR spectra, Raman spectra, and XPS of NiHATP and NiHATP/CNT-1 (Figure S7); SEM image, EDS mapping, IR spectra, Raman spectra, and XPS of Ni­(oPDI)_2_ and Ni­(oPDI)_2_/CNT-1 (Figure S8); NMR spectra of Ni­(oPDI)_2_ and Ni­(oPDI)_2_/CNT-1 (Figure S9) (PDF)
