# Supplementary material for: Enhanced Chemiresistive Sensor Performance through Superior Accessibility to Metal Complex Sites via Triptycene-Skeleton Coordination Nanosheets
Source: ACS Appl Mater Interfaces. 2026 Apr 1;18(14):20890–9. doi: 10.1021/acsami.6c00170 (PMC13088035; doi:10.1021/acsami.6c00170)
Supplement: Supplementary file 1 [file am6c00170_si_001.pdf]

Supporting Information

Enhanced Chemiresistive Sensor Performance through Superior Accessibility to Metal Complex Sites via Triptycene-Skeleton Coordination Nanosheets

Hiroaki Maeda,[1]* Yuta Sudo,[2] Kenji Takada,[1] Naoya Fukui,[1] Hiroshi Nishihara[1,2]*

[1]Research Institute for Science and Technology, Tokyo University of Science
2641, Yamazaki, Noda, Chiba 278-8510, Japan

[2]Graduate School of Science and Technology, Tokyo University of Science
2641, Yamazaki, Noda, Chiba 278-8510, Japan

E-mail: h-maeda@rs.tus.ac.jp (H.M.), nisihara@rs.tus.ac.jp (H.N.)

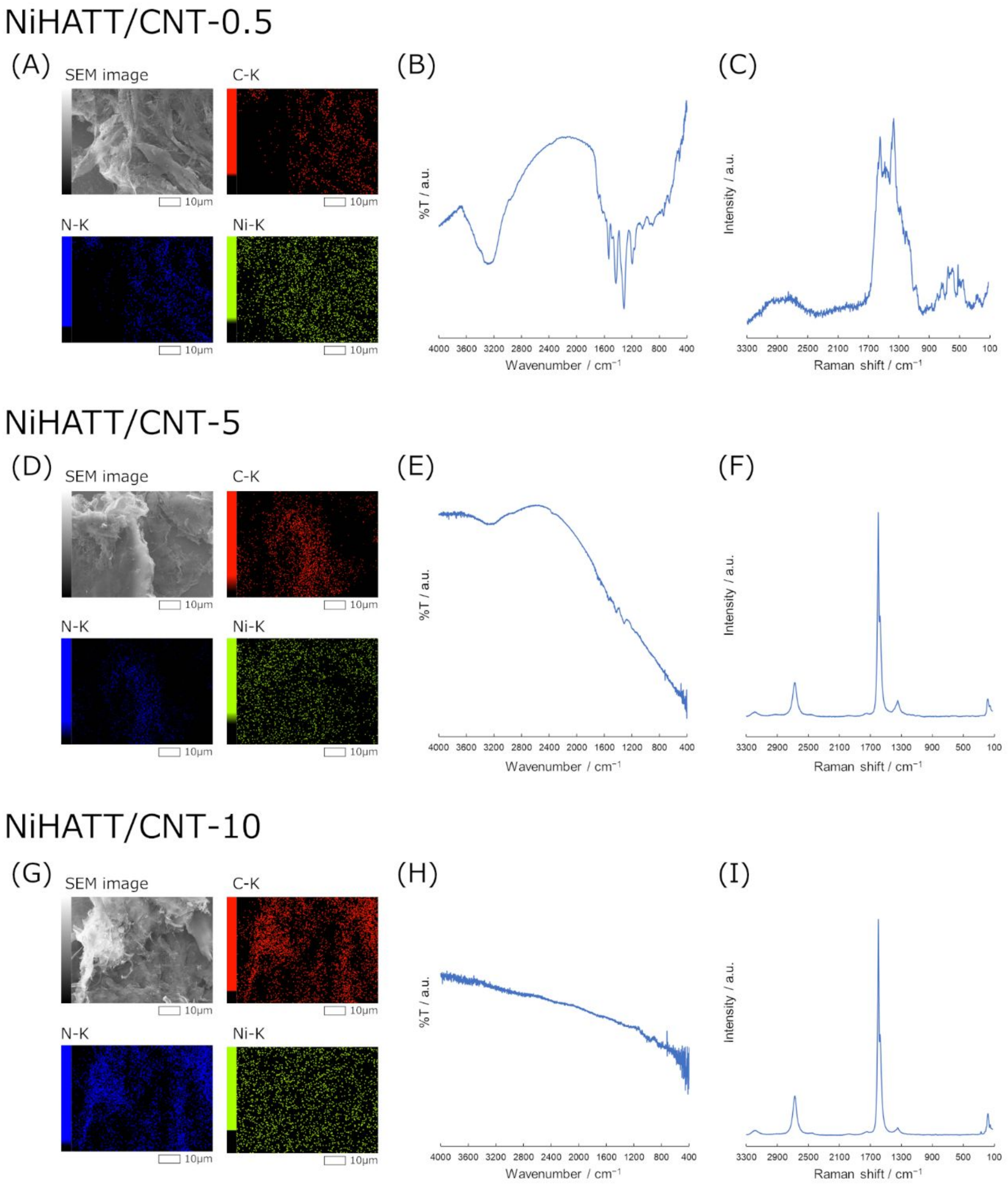

Figure S1. Characterization of NiHATT/CNT-X composites (X = 0.5, 5, and 10). (A, D, G) SEM images and EDS mapping of C, N, and Ni. (B, E, H) IR spectra. (C, F, I) Raman spectra.

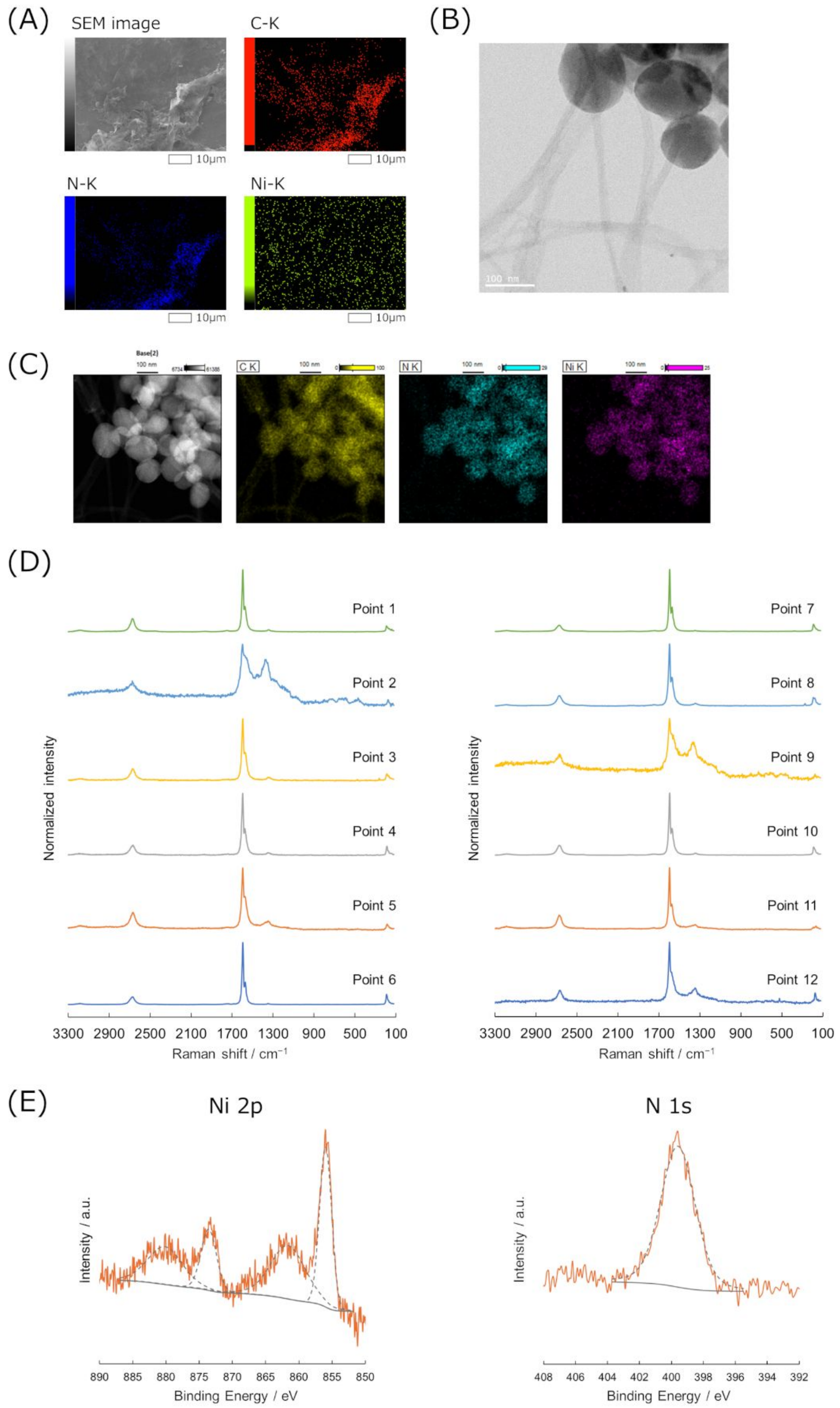

Figure S2. Characterization of NiHATT-CNT mixture. (A) SEM image and EDS mapping of C, N, and Ni. (B) TEM image. (C) TEM image and EDS mapping of C, N, and Ni. (D) Raman spectra recorded at multiple points. (E) XPS spectra of Ni 2p and N 1s regions. Dashed and solid gray lines represent fitting curves and the background line, respectively.

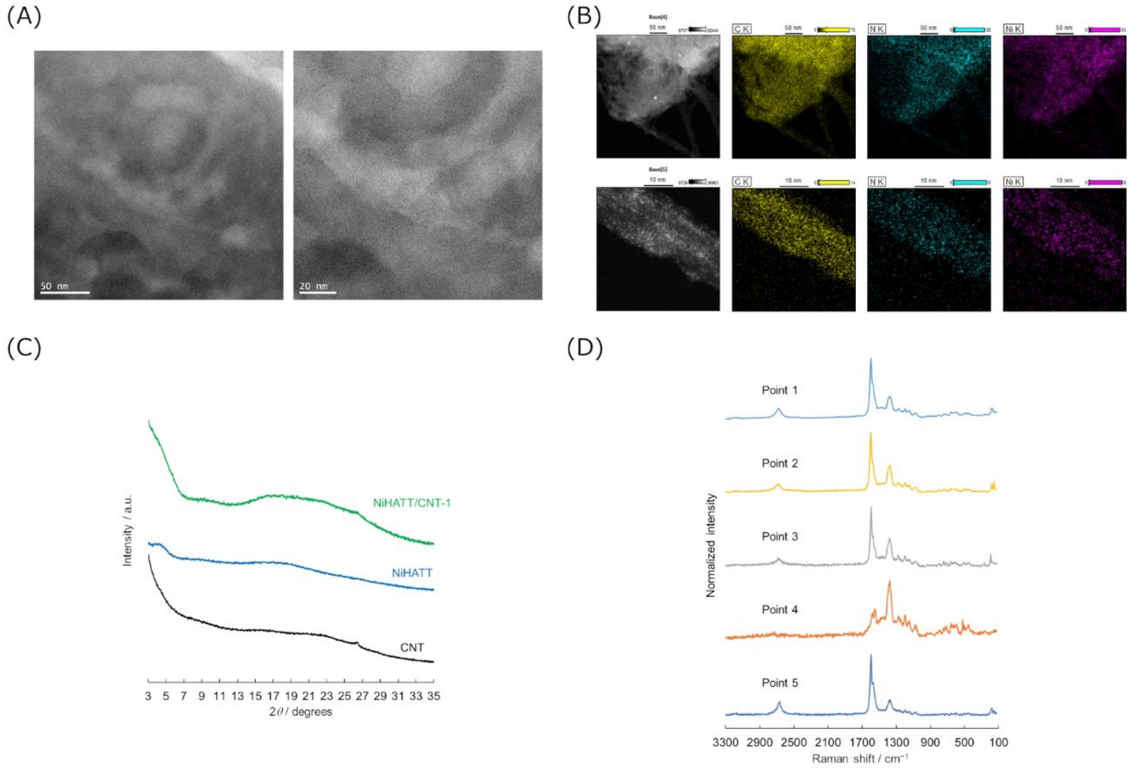

Figure S3. (A) High-resolution TEM images of NiHATT/CNT-1. (B) TEM images and EDS mapping of C, N, and Ni of NiHATT/CNT-1. (C) XRD patterns of NiHATT/CNT-1, NiHATT, and CNT. (D) Raman spectra of NiHATT/CNT-1 recorded at multiple points.

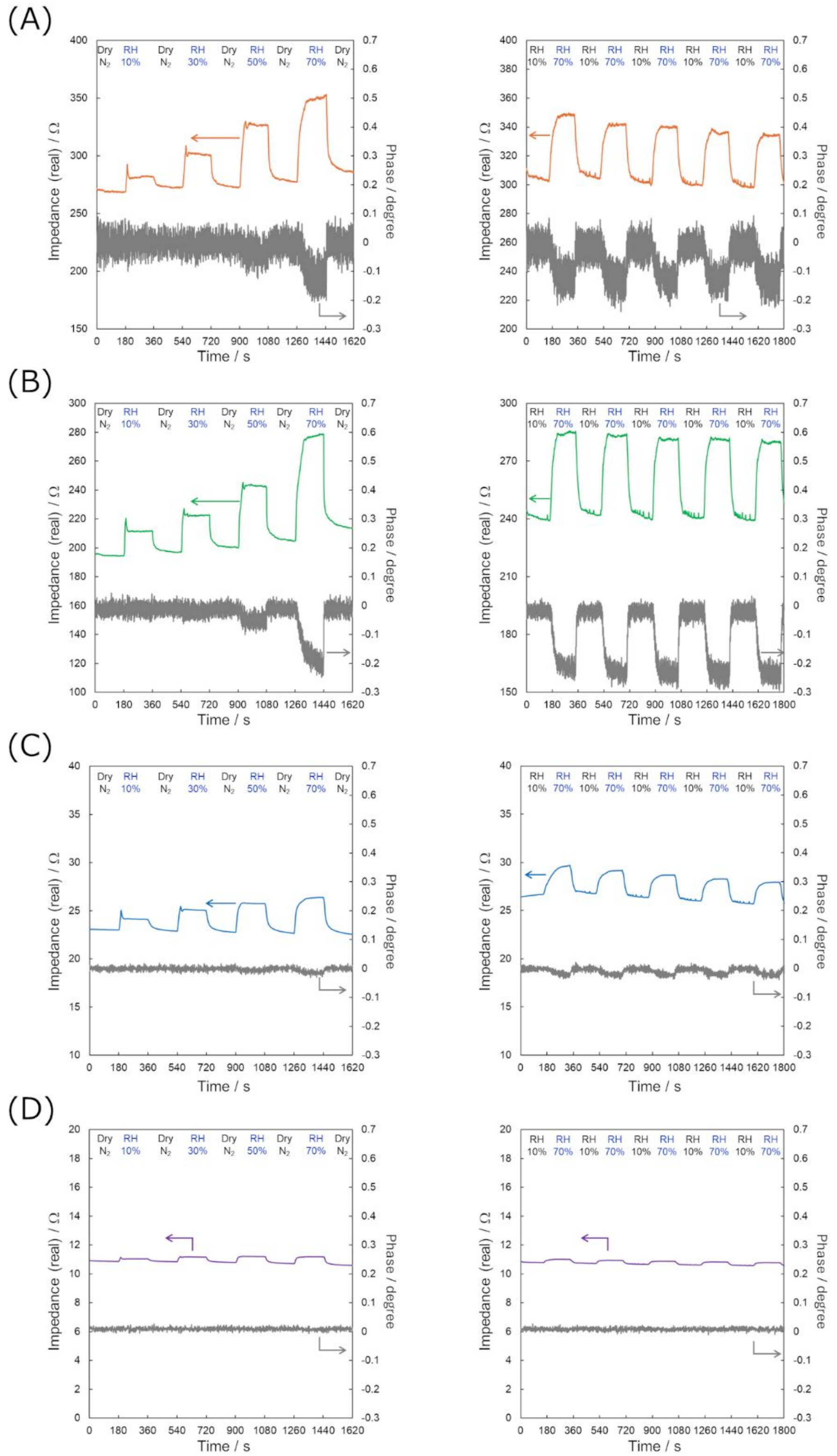

Figure S4. Impedance and phase responses upon exposure to different RH levels in the range of 10-70% and repeated exposure to RH10% and 70% of chemiresistive sensors with NiHATT/CNT-X composites. X = 0.5 (A), 1 (B), 5 (C), and 10 (D).

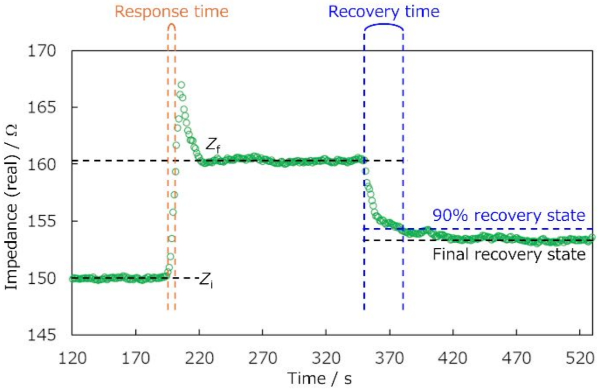

Figure S5. Response and recovery time of NiHATT/CNT-1 at RH 10%.

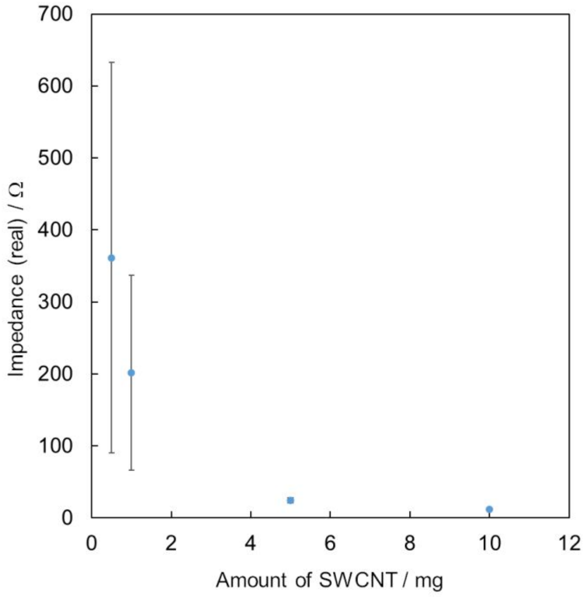

Figure S6. Impedance of chemiresistive sensors with NiHATT/CNT-X composites (X = 0.5, 1, 5, and 10) under dry nitrogen atmosphere.

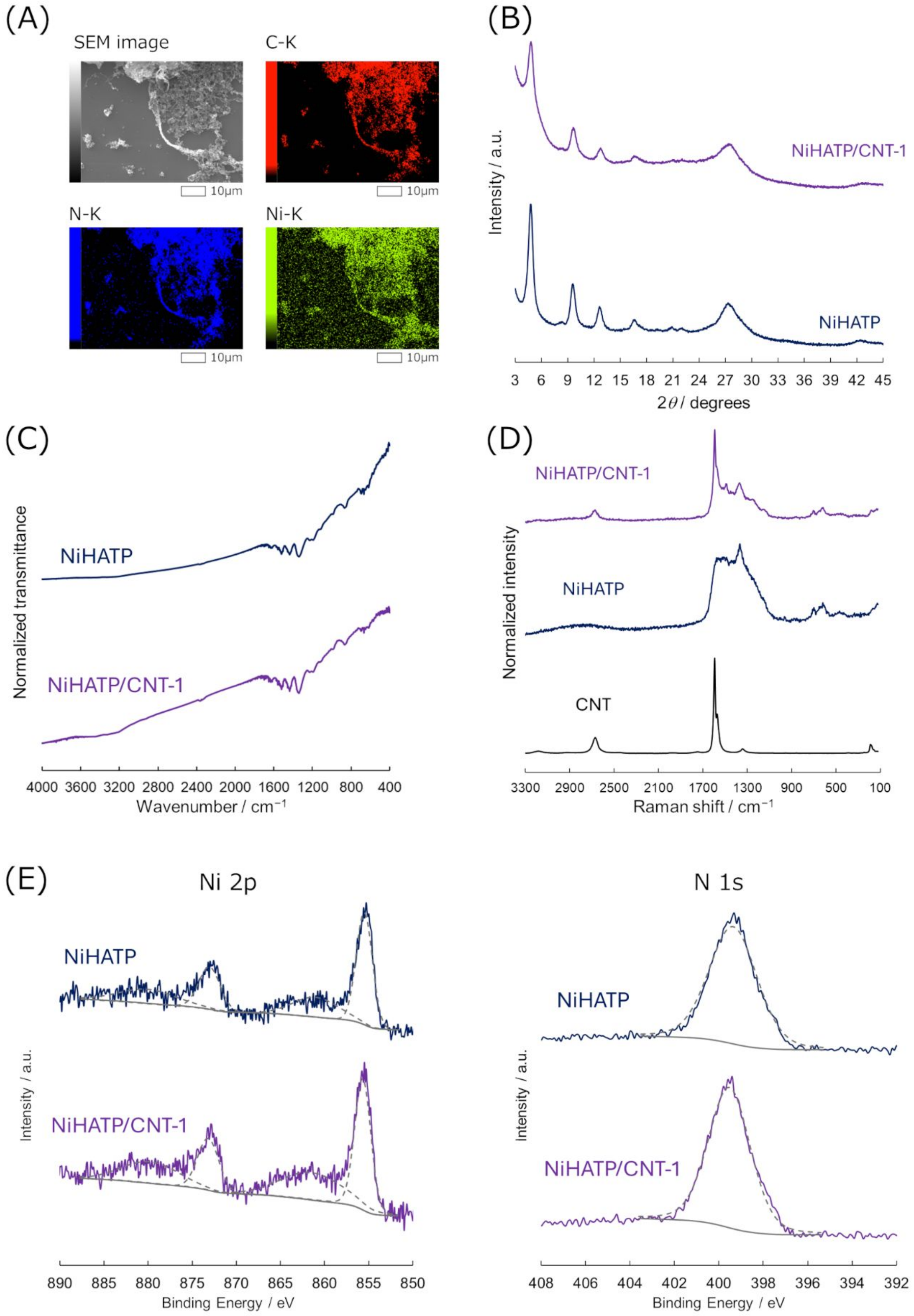

Figure S7. (A) SEM image and EDS mapping of C, N, and Ni of NiHATP/CNT-1. (B) XRD patterns, (C) IR spectra, (D) Raman spectra, and (E) X-ray photoelectron spectra of NiHATP and NiHATP/CNT-1. Dashed and solid gray lines represent fitting curves and the background line, respectively.

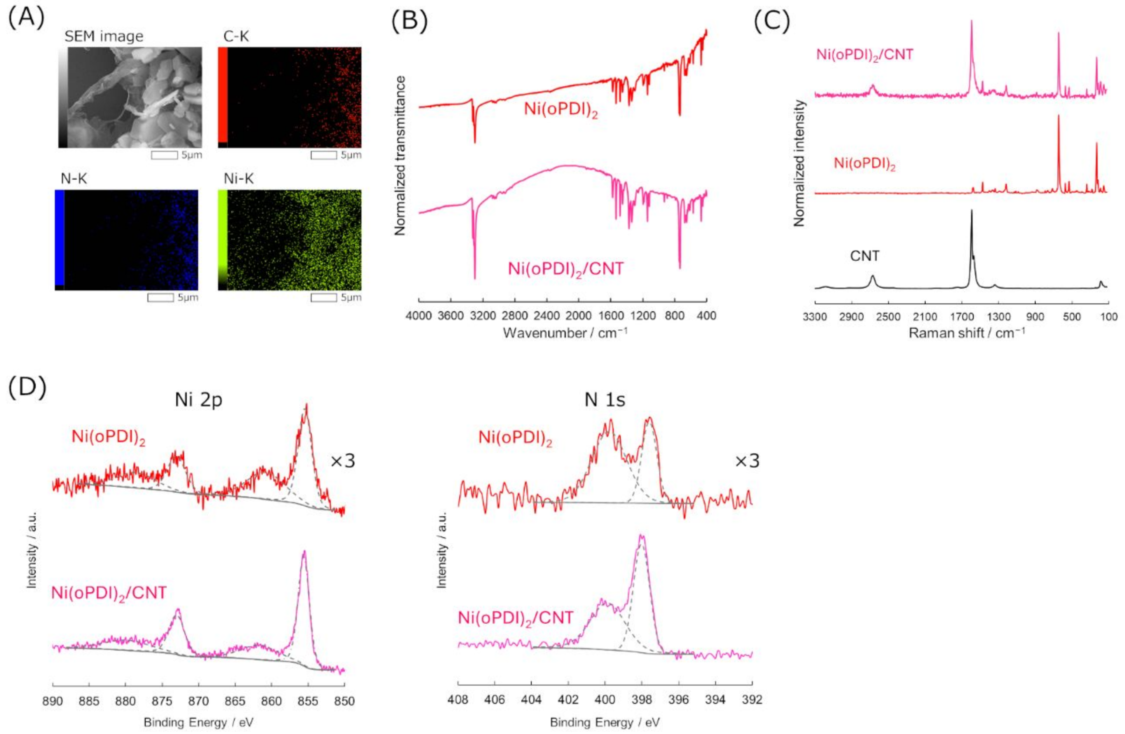

Figure S8. (A) SEM image and EDS mapping of C, N, and Ni of Ni(oPDI)$_2$/CNT-1. (B) IR spectra, (C) Raman spectra, and (D) X-ray photoelectron spectra of Ni(oPDI)$_2$ and Ni(oPDI)$_2$/CNT-1. Dashed and solid gray lines represent fitting curves and the background line, respectively.

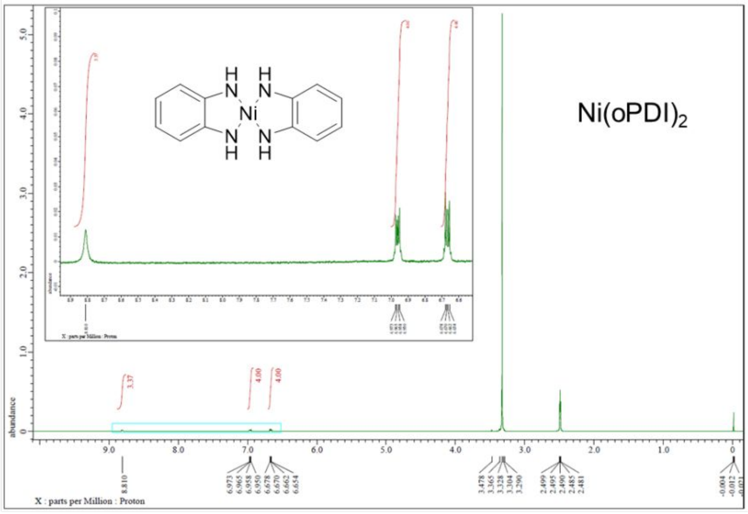

1H-NMR (400 MHz, DMSO-D6) δ 8.81 (s, 4H), 6.96 (dd, 4H), 6.67 (dd, 4H)

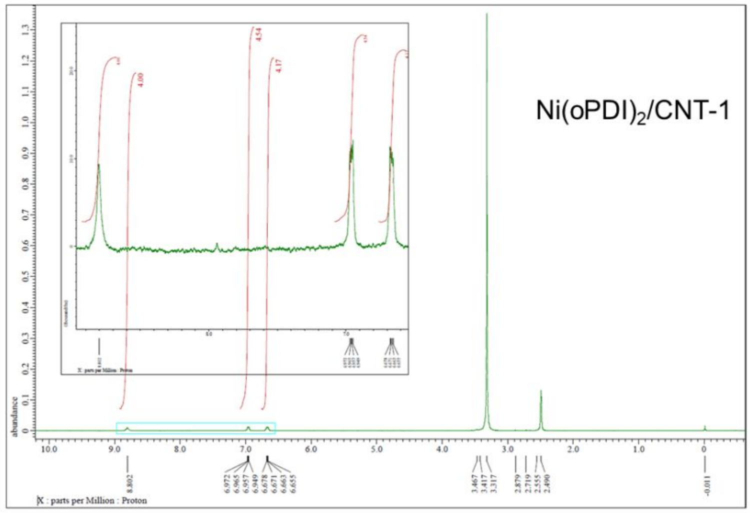

1H-NMR (400 MHz, DMSO-D6) δ 8.80 (s, 4H), 6.96 (dd, 4H), 6.67 (dd, 4H)

Figure S9. ^{1}H NMR spectra of $Ni(oPDI)_2$ and $Ni(oPDI)_2$/CNT-1.
